# Clinical Outcome After Epidural Spinal Cord Stimulation in Patients With Severe Traumatic Brain Injury

**DOI:** 10.7759/cureus.65753

**Published:** 2024-07-30

**Authors:** Alexey N Vorobyev, Aleksandra V Burmistrova, Kiril M Puzin, Maria D Varyukhina, Margarita L Radutnaya, Alexey A Yakovlev, Gennady E Chmutin, Gerald Musa, Egor G. Chmutin, Andrey V Grechko, Gervith Reyes Soto, Carlos Catillo-Rangel, Renat Nurmukhametov, Manuel de Jesus Encarnacion Ramirez, Nicola Montemurro

**Affiliations:** 1 Reanimatology and Rehabilitation, Federal Scientific and Clinical Center for Reanimatology and Rehabilitation, Moscow, RUS; 2 Neurological Surgery, Peoples' Friendship University of Russia, Moscow, RUS; 3 Neurosurgery, Livingstone Central Hospital, Livingstone, ZMB; 4 Neurosurgery, Peoples' Friendship University of Russia, Moscow, RUS; 5 Nervous Diseases and Neurosurgery, Peoples' Friendship University of Russia, Moscow, RUS; 6 Neurosurgical Oncology, National Cancer Institute, Mexico City, MEX; 7 Neurosurgery, ISSSTE Regional Hospital October 1, Mexico City, MEX; 8 Neurosurgery, Azienda Ospedaliera Universitaria Pisana (AOUP) University of Pisa, Pisa, ITA

**Keywords:** post-traumatic brain injury, spinal cord, chronic disorders of consciousness, spastic syndrome, epidural spinal cord stimulation (escs)

## Abstract

Introduction: Epidural spinal cord stimulation is a minimally invasive procedure with a growing list of indications. It has a good safety profile and analgesic effect, reduces the severity of spasticity, and activates various brain regions. The purpose of this study is to evaluate the clinical outcome of epidural spinal cord stimulation in patients with spastic syndrome and chronic disorders of consciousness resulting from severe traumatic brain injury (sTBI).

Methods: Between 2021 and 2023, an epidural spinal cord stimulation test was performed in 34 patients with central paresis, severe hypertonia, and chronically altered consciousness following sTBI. The severity of spastic syndrome was assessed using a modified Ashworth scale. All patients underwent implantation of a cylindrical eight-contact test epidural electrode at C3-C5 cervical level, followed by neurostimulation and selection of individual modes. Tonic stimulation at a frequency of 60 Hz, "burst" mode, or a combination of the two was used.

Results: Epidural spinal cord stimulation was administered for an average of 4 ± 1.5 days, with tonic stimulation mode applied in 15 (44.1%) patients, "burst" mode in 10 (29.4%), and a combination of two in nine (26.5%) patients. A reduction in spasticity with clinical improvement was observed in 21 patients (61.8%). The Ashworth scale scores for distal and proximal upper extremities decreased from 3 points to 2.5 points and from 3 points to 2 points, respectively. This was significant in the right upper limbs (p = 0.0152 distally and p = 0.0164 proximally). Significant improvements were also seen in the lower extremities. Active movements in paretic limbs increased or appeared in 12 patients (35.3%), while a heightened level of consciousness was observed in six patients (17.6%). Permanent neurostimulator implantation was performed in 12 patients (35.3%), with no reported surgical complications.

Conclusion: Epidural spinal cord stimulation shows promise as an invasive rehabilitation method for patients with sTBI sequelae. Its use reduced the severity of spastic syndrome in over half of patients and increased active movements in paretic limbs in over a third. Notably, neuromodulation at the cervical level yielded pronounced effects on the upper extremities, both proximally and distally. Findings regarding consciousness level improvement are particularly intriguing but warrant further validation through randomized trials.

## Introduction

Traumatic brain injury (TBI) represents a critical challenge in contemporary medicine both globally and locally, due to its extensive prevalence, significant risk of temporary and permanent disability, and cause of mortality worldwide, accounting for about 30% of all injury-related deaths [1,2] and a notably high incidence among young and middle-aged individuals - the most active segment of the workforce and social milieu [3]. In Russia, trauma ranks as the second leading cause of mortality and a major contributor to disability [3]. Epidemiological studies highlight the alarming frequency of TBI in Russia, with approximately 600,000 cases annually. Of these, around 50,000 individuals succumb to their injuries, and an equivalent number are officially recognized as disabled [3-5]. Upon hospital admission, approximately 62.8-73.2% of TBI victims are in satisfactory overall condition. Among them, 18.2-20.7% are categorized as having moderate severity, while 4.2-7.9% exhibit severe or extremely severe conditions, with a negligible fraction classified as terminal, at 0.1-1.9% [6]. Various authors have provided statistical insights into the spectrum of impaired consciousness observed in TBI survivors during hospitalization, ranging from clear consciousness in 70.4-79.7% to varying degrees of stupor and coma [6,7]. Post-trauma, spastic syndrome emerges in 59-70% of TBI cases within a year and may persist in up to 89% of cases over the long term [8,9].

The management of spastic syndrome continues to be a subject of global investigation [10,11]. Treatment modalities encompass diverse approaches, such as drug therapy, botulinum toxin type A injections into affected limbs, intrathecal baclofen administration, and surgical interventions, including selective dorsal rhizotomy. However, the latter carries risks of complications such as hypersensitivity, muscle spasms, and gait disturbances [12-14]. In recent decades, the field of neurosurgery has increasingly turned its attention to epidural spinal cord stimulation as a potential solution for spastic syndrome. This minimally invasive procedure offers several advantages, including relative safety, minimal surgical intervention, low complication rates, analgesic effects, and the capacity to ameliorate pathological muscle tone while activating various brain regions [15-17]. Nevertheless, the impact of spinal cord neurostimulation on enhancing consciousness levels post-TBI remains inadequately understood [18].

## Materials and methods

Study design and patient cohort

This retrospective study evaluates the outcomes of assessments and surgical interventions performed on a cohort of 34 patients undergoing rehabilitation at the Research Institute of Rehabilitation, Russian National Research Medical Center, between 2021 and 2023. The patients, aged 20-48 years, exhibited sequelae from severe traumatic brain injury (TBI), which means a TBI with a GCS ≤ 8 at first presentation in the emergency room.

The primary objective of this paper is to evaluate the short-term clinical outcome of these patients, particularly in spastic syndrome and in the state of consciousness, after epidural spinal cord stimulation. A short-term follow-up of seven days was reported.

Inclusion criteria are patients with age > 18, both, spastic syndrome (2-4 according to the modified Ashworth scale [19]) and/or chronic disorders of consciousness resulting from a severe TBI, treated with epidural spinal cord stimulation between 2021 and 2023. A comprehensive comparative analysis was conducted among the patients, considering variables such as gender, age, and disease severity, including the extent of spastic syndrome and the degree of impaired consciousness.

The study was conducted in accordance with the principles of biomedical ethics formulated in the Helsinki Declaration of 1964 and approved by the local bioethical Committee of the Federal Research and Clinical Center for Intensive Care and Rehabilitation, Moscow (protocol no. 19.01.12). All participants provided voluntary written informed consent, signed by the participant or their representative, to publish any potentially identifiable images or data included in this paper.

Surgical technique

**Figure 1:**
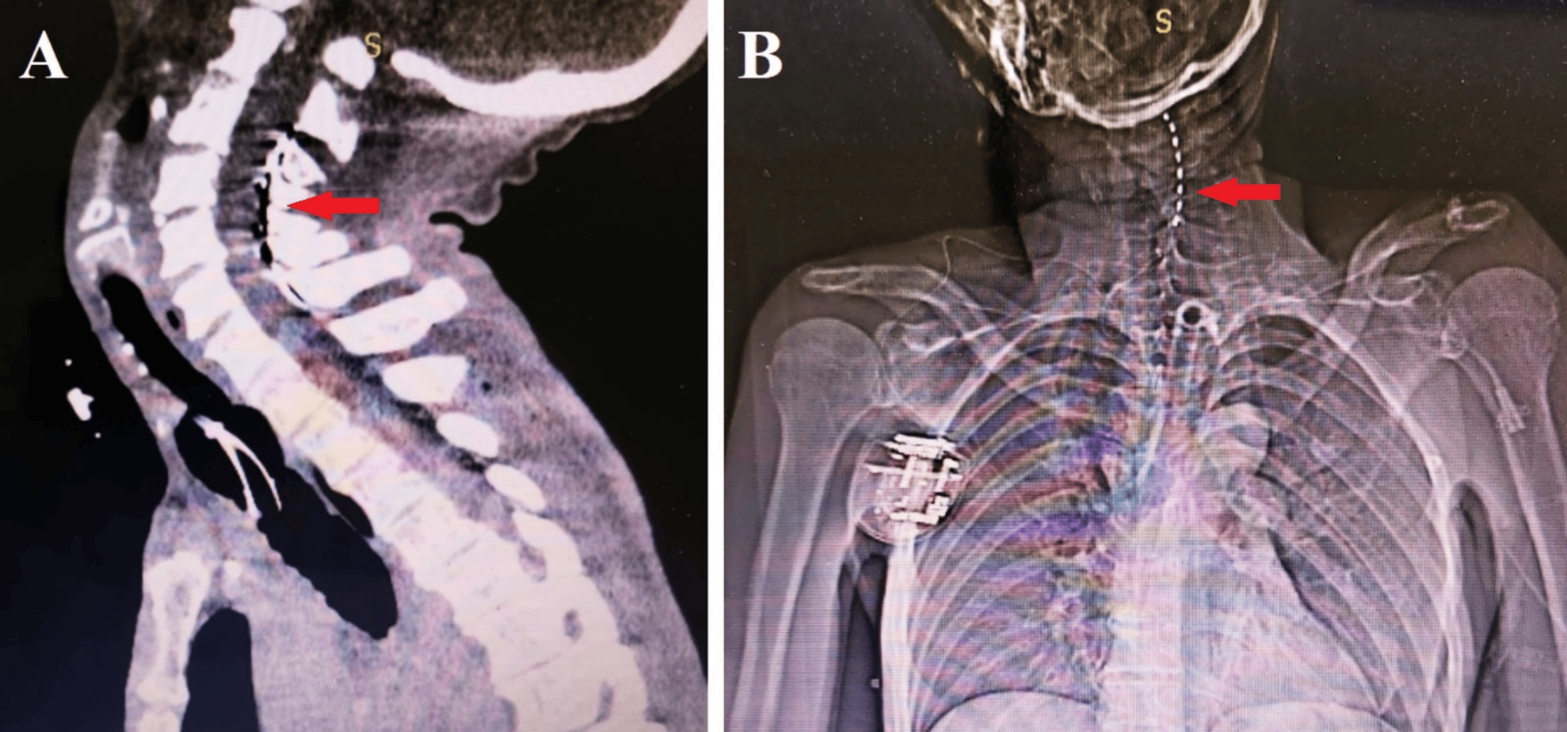
Postoperative CT (A) and X-ray (B) showing the epidural spinal cord electrode (red arrows) at the C3-C5 level.

All patients underwent surgical implantation of a test epidural eight-contact cylindrical electrode to stimulate the spinal cord at the C3-C5 level. The procedure was conducted under total intravenous anesthesia with mechanical ventilation administered via an orotracheal tube with the patient positioned on his side. Intravenous propofol emulsion was used as the primary anesthetic agent at calculated doses, while analgesia was achieved through intermittent intravenous administration of fentanyl solution (1-2 mL of 0.005%) with repeated dosing every 20-25 minutes. Muscle relaxation was facilitated by administering rocuronium bromide solution in calculated doses. Myoplegia was essential to ensure precise electrode placement within the epidural space, thereby minimizing the risk of dislocation and intraoperative complications. Under the guidance of an electron-optical transducer, the eight-contact electrode was positioned within the epidural space at the C3-C5 level, with the distal end specifically placed at the C3 vertebra level. Following surgery, cervical spine CT scans were performed on all patients to verify electrode positioning and rule out potential intraoperative complications (Figure 1). In two cases, stimulation selection was performed under needle myography control in the operating room upon cessation of muscle relaxant effects.

Postoperative epidural spinal cord stimulation

**Figure 2:**
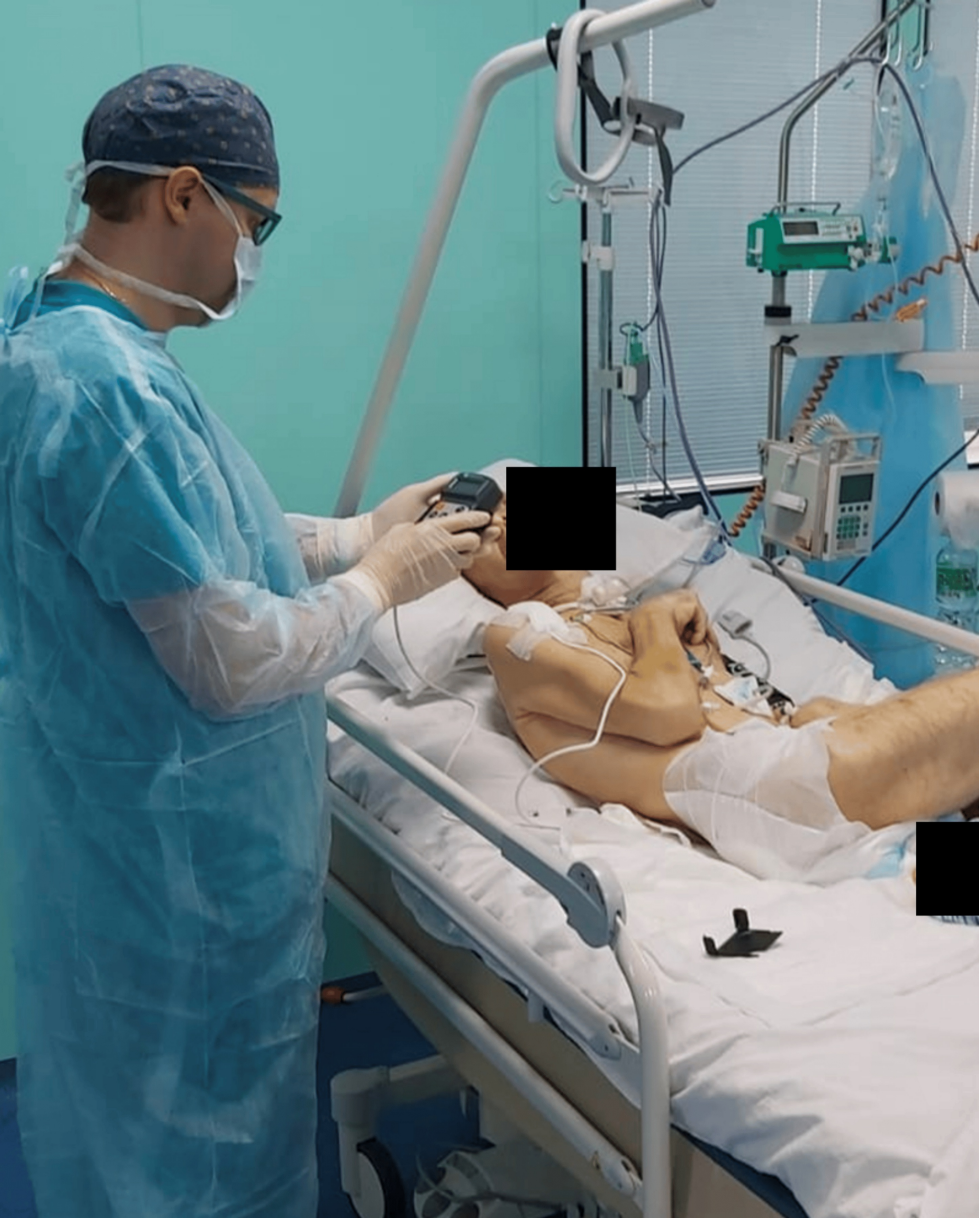
Epidural spinal cord stimulation during the postoperative period.

On the subsequent postoperative day, all patients underwent epidural spinal cord stimulation with individualized mode customization (Figure 2), including adjustments in frequency, pulse width, amplitude, and electrode charge. Various stimulation modes, including tonic stimulation, "burst" mode, or combinations thereof, were employed based on the achieved clinical effects. Tonic mode parameters were set with a pulse duration of 200-250 microseconds and a stimulation frequency of 60 Hz, while burst mode featured a pulse duration of 1,000 microseconds (fivr pulses) at a frequency of 40 Hz. The current amplitude for testing initially commenced at 1 mA and was gradually increased until patients experienced paresthesia, limb tremors, or signs of pain based on the behavioral pain scale. Subsequently, current amplitudes were adjusted to patient comfort levels, ensuring the absence of discomfort or pain. Electrode impedance, indicative of electrical resistance, was measured using a pulse generator program analyzer, with all cases achieving acceptable values.

Neurological assessments

Neurological assessments were conducted in both pre- and postoperative (seven days later) periods by a single neurologist, evaluating the severity of the spastic syndrome and the level of consciousness using the modified Ashworth scale [19], the Glasgow coma scale [20], and the revised coma recovery scale (CRS-R) [21]. The modified Ashworth scale is the most universally accepted clinical tool used to measure the increase of muscle tone, where 0 means no increase in muscle tone and 4 means affected parts rigid in flexion or extension. Additionally, myographic monitoring was employed to evaluate stimulation effectiveness using myographic electrodes.

Statistical analysis

Descriptive statistics were employed to summarize quantitative data, with normally distributed indicators, such as age, presented as mean ± standard deviation. For non-normally distributed or skewed indicators, the data were reported as median. Dichotomous variables were expressed as absolute and relative frequencies (fractions). The Spearman test was utilized to identify correlations between variables both pre- and post-surgery. A critical significance level of 0.05 was employed for statistical tests. Statistical analyses were performed using the STATISTICA (version 10; StatSoft Inc., Tulsa, OK) software package.

## Results

The study cohort comprised 34 individuals with an average age of 34 ± 14 years and a male-to-female ratio of 2.4:1. Central paresis accompanied by pronounced musculotonic syndrome was universally observed, with tetraparesis in 22 patients (64.7%), hemiparesis in nine patients (26.5%), and spastic monoparesis in three patients (8.8%). Then, the mean GCS and the mean modified Ashworth scale were 9.1 and 3 points, respectively. Table 1 shows all the details.

**Table 1:** Study population.

	n (%)
Patients	34
Mean age (years)	34 ± 14
Male:female	2.4:1
Clinical presentation	
Tetraparesis	22 (64.7%)
Hemiparesis	9 (26.5%)
Spastic monoparesis	3 (8.8%)
GCS at first presentation	
8	11
7	13
6	7
5	3

The severity of spastic syndrome was quantified using the modified Ashworth scale. Prior to epidural spinal cord stimulation, chronic disorders of consciousness were identified in 20 patients (58.8%), with five patients (14.7%) in a vegetative state, five patients (14.7%) in a state of minimal consciousness minus, nine patients (26.5%) in a state of minimal consciousness plus, and one patient (2.9%) in emerging consciousness state.

**Figure 3:**
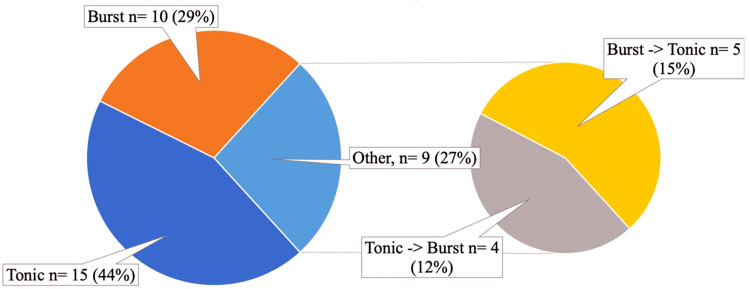
Distribution of the stimulation regimes.

The average duration of epidural spinal cord stimulation was 4 ± 1.5 days. Among the stimulation regimens employed, tonic stimulation was utilized in 15 patients (44.1%), "burst" mode in 10 patients (29.4%), and a combination of modes in nine patients (26.5%) (Figure 3).

**Figure 4:**
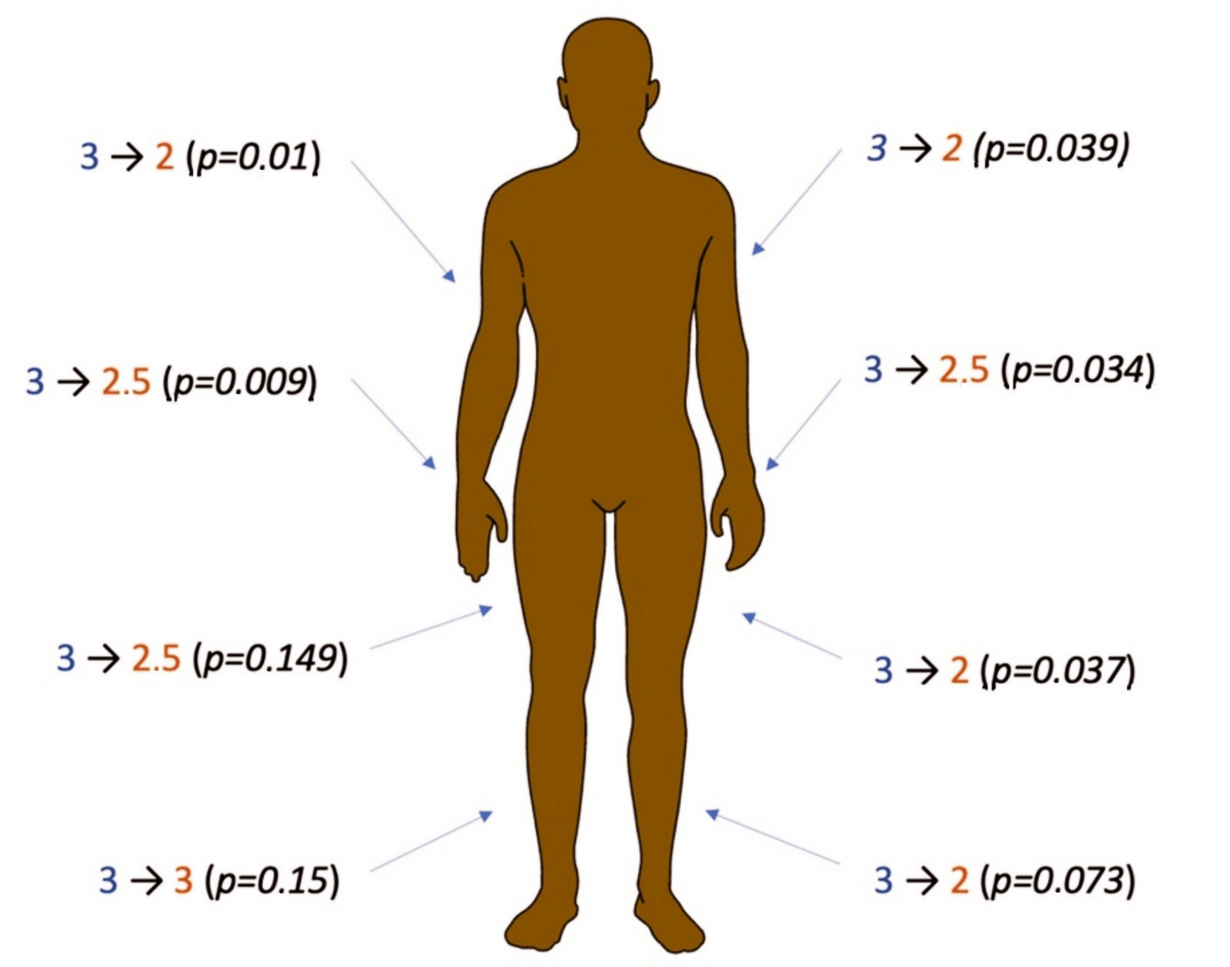
Pre- and post-stimulation spasticity severity according to the Ashworth scale. This figure also shows a reduction in the severity spastic in all areas of the body.

During stimulation, a significant reduction in spastic syndrome severity was observed in 21 patients (61.8%). Specifically, the median score on the modified Ashworth scale decreased from 3 points to 2.5 points for distal parts of the upper extremities and from 3 points to 2 points for proximal parts. Notably, the most pronounced reduction in spasticity occurred in the right upper limb (p = 0.009 for distal muscles and p = 0.0164 for proximal muscles), with no statistically significant differences observed in the lower extremities (Figure 4).

According to the modified Ashworth scale [19], active movements in the paretic limbs increased or emerged in 12 patients (35.3%). In addition, according to CRS-R [21], an increase in consciousness level, manifested as enhanced patient interaction and emotional responsiveness, was reported in six patients (17.6%). Following the stimulation course, 12 patients (35.3%) underwent implantation of a permanent neurostimulator. Notably, no surgical complications were encountered throughout the entire process, including surgery and stimulation.

## Discussion

The application of epidural spinal cord stimulation at the cervical level among patients with severe TBI injury during the rehabilitation phase yielded promising results, notably in reducing spasticity and potentially enhancing consciousness levels within the studied cohort. These findings indicate a significant advancement in both motor and cognitive rehabilitation strategies tailored to this patient population.

Our study's outcomes align with existing research on the efficacy of epidural spinal neuromodulation for individuals with spasticity and chronic consciousness disorders. For instance, Wu et al. [22] conducted a comprehensive meta-analysis of 10 studies involving 508 patients using spinal cord stimulation for chronic consciousness disorders and spasticity. Their findings revealed an average effectiveness rate of 37%, with 30% of patients experiencing heightened consciousness levels following cervical-level spinal cord stimulation, particularly among those with reactive wakefulness syndrome. Although this analysis encompasses a substantial number of patients, variations in stimulation parameters, duration, and implantation sites across studies necessitate cautious interpretation. Furthermore, Jung et al. reviewed 43 studies examining spinal cord stimulation in patients with spasticity, concluding that the method is both safe and effective, with efficacy rates ranging from 40% to 78% [23].

While our study's results are consistent with existing literature, they warrant further scrutiny through rigorous statistical analysis on a larger sample size. This is particularly pertinent concerning improvements in patients' consciousness levels [24-26]. One plausible mechanism underlying this phenomenon could be the formation of transient neural networks induced by the ascending effects of stimulation, as evidenced in our prior study utilizing functional magnetic resonance imaging (fMRI) in patients undergoing spinal cord stimulation [27,28]. These transient networks may facilitate enhanced neural plasticity and synaptic connectivity, contributing to improvements in motor and cognitive functions.

The integration of 3D technology into epidural spinal cord stimulation procedures significantly enhances preoperative planning and precision in electrode placement. High-resolution imaging techniques, such as MRI and CT scans, can be converted into detailed 3D models of the patient’s spinal anatomy [29,30]. These models allow surgeons to visualize and plan the exact placement of epidural electrodes, ensuring accurate targeting of the desired spinal cord regions. This precision minimizes the risk of complications and maximizes the therapeutic benefits of epidural spinal cord stimulation [31,32]. 3D technology facilitates real-time surgical navigation through augmented reality (AR) and virtual reality (VR) systems. Surgeons can overlay 3D anatomical models onto the patient's body during surgery, providing a precise roadmap for electrode placement [33]. This augmented visualization aids in avoiding critical structures and enhances the accuracy of electrode positioning, which is crucial for the effectiveness of spinal cord stimulation in reducing spasticity and improving motor functions [34,35].

The use of 3D printing allows for the creation of patient-specific electrodes tailored to the unique anatomical features of each patient. Custom electrodes can conform more closely to the spinal cord’s contours, improving the efficiency and consistency of electrical stimulation. This customization ensures optimal contact between the electrodes and the spinal cord, leading to better clinical outcomes and reduced spasticity [30,31,36]. 3D-printed models serve as valuable tools for training neurosurgeons in epidural spinal cord stimulation techniques. These models replicate the complex anatomy of the spinal cord and surrounding tissues, allowing surgeons to practice and refine their skills in a risk-free environment. Such training enhances the proficiency and confidence of surgeons, contributing to improved patient outcomes and reduced intraoperative errors [37-39].

After the implantation of epidural electrodes, 3D imaging can be used to assess the placement and function of the electrodes [32,40]. This assessment allows for precise postoperative adjustments to the stimulation parameters, ensuring that the therapeutic goals are met. The ability to visualize and adjust the electrodes in three dimensions enhances the overall effectiveness of the treatment [41-44].

Limitations of the study

Despite the promising results of epidural spinal cord stimulation in patients with severe TBI, this study has several limitations. The study included a relatively small cohort of 34 patients, which limits the generalizability of the findings. Larger studies with more diverse populations are necessary to validate the results and ensure that they can be universally applied.

The average duration of epidural spinal cord stimulation was 4 ± 1.5 days, and follow-up periods were not extensively detailed. Longer follow-up periods are essential to assess the long-term efficacy and safety of epidural spinal cord stimulation in these patients. The study design did not include randomized controlled trials (RCTs), which are considered the gold standard for determining the efficacy of a treatment. Future studies should incorporate RCTs to provide more robust evidence of the benefits of epidural spinal cord stimulation.

There was variability in the stimulation modes and parameters used (tonic stimulation, "burst" mode, and combinations), which might affect the outcomes. Standardizing these parameters in future studies would help in drawing more consistent and reliable conclusions. Although some improvement in consciousness levels was observed, the mechanisms behind these changes were not fully explored. Further research using advanced neuroimaging and electrophysiological monitoring is needed to understand how epidural spinal cord stimulation affects consciousness.

The study was conducted at a single center, which may introduce bias related to specific clinical practices and patient demographics. Multi-center studies would provide a more comprehensive evaluation of the intervention across different settings and populations. Neurological assessments were conducted by a single neurologist, which could introduce observer bias. Including multiple assessors and using blinded assessment protocols would enhance the objectivity of the results.

Moreover, while our study suggests potential benefits in consciousness enhancement, further research employing rigorous methodologies, including RCTs, is essential to validate these findings and elucidate underlying mechanisms comprehensively. Such trials should incorporate advanced neuroimaging techniques and electrophysiological monitoring to explore the specific neural pathways and mechanisms activated by cervical epidural stimulation.

## Conclusions

Cervical-level epidural spinal cord stimulation represents a promising therapeutic intervention for reducing spasticity and potentially enhancing consciousness in patients with severe TBI. Our findings add to the growing body of evidence supporting neuromodulation in neurorehabilitation. Notably, this intervention reduced spasticity severity in over half of the patients and increased active movements in paretic limbs in over a third, with significant effects on the upper extremities. These promising results, particularly regarding consciousness improvement, warrant further validation through larger cohorts and RCTs with standardized protocols to fully elucidate the therapeutic potential and mechanisms of this intervention.
